# Interplay Among Classroom Environment, Grit, and Enjoyment in Shaping Feedback-Seeking Behavior in L2 Writing

**DOI:** 10.3390/bs15050584

**Published:** 2025-04-27

**Authors:** Wenqian Luan, Jianqiang Quan

**Affiliations:** School of Foreign Languages, East China Normal University, Dongchuan Road No. 500, Shanghai 200241, China; 71264800190@stu.ecnu.edu.cn

**Keywords:** writing feedback-seeking behavior, foreign language enjoyment, L2 grit, perseverance of effort, consistency of interest, classroom psychological environment, positive psychology

## Abstract

The interplay among classroom environment, grit, and enjoyment in shaping the feedback-seeking behavior (FSB) of Chinese English as a Foreign Language (EFL) learners remains underexplored. This study investigates how the classroom psychological environment and L2 grit influence FSB, categorized as feedback monitoring (FM, the passive observation of feedback) and feedback inquiry (FI, proactive requests for clarification), in the context of L2 writing. This study also focuses on the mediating role of foreign language enjoyment (FLE) in this process. A mixed-methods design was utilized to study 612 Chinese junior secondary students aged 13–15 with over five years of formal English instruction. Structural equation modeling (SEM) revealed that perseverance of effort (POE) and consistency of interest (COI), as two dimensions of L2 grit, directly predicted FM (β = 0.19 and 0.27, respectively) but not FI. The classroom environment indirectly enhanced both FM (β = 0.05) and FI (β = 0.09) through FLE. Qualitative interviews highlighted cultural constraints: 83.3% of participants prioritized FM over FI due to face-saving norms, despite high grit levels (M = 3.61 on a 5-point scale), underscoring cultural barriers to proactive feedback-seeking in Chinese collectivist classrooms. These findings validate the tripartite framework of positive psychology in L2 learning and propose strategies to balance institutional support, grit cultivation, and cultural sensitivity in fostering adaptive FSB.

## 1. Introduction

Over the past two decades, written corrective feedback (WCF) has emerged as a pivotal yet contentious topic in L2 writing research (Chandler, 2003; Ferris, 2010). While substantial evidence supports its effectiveness in improving linguistic accuracy (Bitchener, 2008), persistent discrepancies in findings reveal critical limitations (Truscott & Hsu, 2008). Notably, traditional WCF frameworks often treat learners as passive recipients, rather than active agents (Papi et al., 2020). This systematic oversight of their subjective engagement in feedback processes has prompted a paradigm shift toward investigating FSB, where learners proactively solicit and process feedback.

Studies on the influencing factors of FSB in L2 writing are still in the early stages of investigation, which mostly concentrate on the degree of individual factors like language mindsets, goal orientations, self-efficacy, L2 writing ego, and shyness (Papi et al., 2020; Papi et al., 2019; Xu & Wang, 2024; Yao & Zhu, 2024; Zhang, 2024). Research examining the combined effects of institutions, emotions, and personality traits on L2 FSB remains limited and fragmented (Zhang, 2024; Zhang & Jiang, 2025) and is critically constrained by three persistent gaps. First, while existing studies have predominantly emphasized negative emotions (e.g., anxiety and shyness) as inhibitors of FSB (Xu & Wang, 2024; Zhang & Jiang, 2025), they have neglected the facilitative role of positive emotions, such as FLE—a significant oversight given the growing recognition of positive psychology in SLA (Guo & Qiu, 2022). Second, environmental influences have been narrowly conceptualized as teacher support (Zhang, 2024; Zhang & Jiang, 2025), failing to account for the multidimensional classroom dynamics that may critically shape feedback-seeking decisions in collectivist learning contexts. Third, methodological approaches remain imbalanced, favoring quantitative designs while lacking qualitative insights into learners’ cognitive–emotional processes during FSB.

This study is guided by the goal of advancing FSB theory and practice, and adopts Seligman and Csikszentmihalyi’s (2000) tripartite mode. This mode synthesizes institutional (classroom psychological environment), personal (L2 grit, comprising COI, defined as sustained commitment to long-term language goals, and POE, reflecting proactive resilience despite obstacles), and emotional (FLE) factors. This integrative framework addresses a critical limitation in prior research, which has predominantly examined these variables in isolation (Dewaele & MacIntyre, 2014; Zhao & Wang, 2023b). Furthermore, it introduces cultural considerations by theorizing how “face” norms, defined as learners’ strategic negotiation of social dignity and relational harmony in collectivist classrooms, may influence the relationship between classroom environments and FSB, particularly in contexts where public error correction risks social embarrassment. Finally, it employs a mixed-methods design to triangulate quantitative structural equation modeling with qualitative interviews, addressing methodological imbalances in prior research.

Guided by these objectives, this study proposes three research questions:

To what extent do L2 grit (comprising POE and COI) and the classroom psychological environment collectively predict FSB in L2 writing?

How does FLE mediate the relationships among L2 grit, classroom environment, and FSB?

In addition to the above, what other factors influence learners’ FSB within the proposed mediation framework?

This research addresses these questions. In doing so, it advances the theoretical understanding of FSB through a positive psychology lens. It also offers practical strategies, which aim to cultivate feedback-rich L2 writing environments that honor learners’ cultural and emotional needs. The remainder of this paper is structured as follows: Section 2 reviews the literature on FSB, grit, classroom environments, and FLE; Section 3 outlines the mixed-methods design; Section 4 and Section 5 present the quantitative and qualitative findings; Section 6 discusses the implications; and Section 7 concludes with limitations and future directions.

## 2. Literature Review

### 2.1. L2 Writing Feedback-Seeking Behavior (FSB)

FSB, initially conceptualized by Ashford and Cummings (1983), delineates the process through which individuals actively observe and pursue feedback in alignment with organizational objectives and personal aspirations. In investigations of WCF, researchers have identified that learners‘ individual variations and motivational drivers not only affect their receptivity to feedback, but also critically shape their engagement in feedback-related activities. Consequently, the organizational psychology construct of FSB has been integrated into second language acquisition (SLA) research to emphasize learners’ agentic roles in feedback interactions and examine its motivational underpinnings (Papi et al., 2019). FSB comprises two dimensions: feedback monitoring, an implicit process involving attention to feedback cues, and feedback inquiry, an explicit process entailing direct requests for externally generated feedback (Xu & Wang, 2023).

Research on factors influencing FSB in L2 writing has developed dynamically, with existing findings emphasizing individual cognitive frameworks. Early studies were centered on contrasting learner mindsets. Individuals with a growth mindset strategically utilize monitoring and inquiry approaches to acquire feedback (Papi et al., 2020; Papi et al., 2019). Conversely, fixed-mindset learners, limited by concerns over self-presentation costs, confine feedback exchanges to trusted instructors in low-risk contexts (Yao & Zhu, 2024). This distinction gains clarity through achievement goal theory, which posits that growth mindsets correlate with mastery goals to foster adaptive feedback cycles, while fixed mindsets show nuanced links to performance goals. Specifically, performance-approach goals may motivate authority-focused feedback-seeking behaviors, whereas performance-avoidance goals strongly inhibit FSB (Elliot & Mcgregor, 2001; Papi et al., 2019). Both academic buoyancy (Xu & Wang, 2023) and ideal L2 writing self-concept (Zhan et al., 2023) are positive predictors of feedback monitoring and feedback inquiry, suggesting that more motivated students seek out feedback. Shyness and the self-regulatory component of self-efficacy predict feedback monitoring, whereas self-efficacy predicts feedback inquiry (Xu & Wang, 2024). Sporadic studies have preliminarily advanced our understanding of environmental determinants of FSB in L2 writing. Zhang (2024) established that teacher academic support not only directly predicts FSB, but also exerts indirect effects through mediating mechanisms involving the ideal L2 writing self-concept. Building on this foundation, Zhang and Jiang (2025) employed structural equation modeling to demonstrate that within Chinese higher education systems, teacher support directly enhances FM and FI. Their analysis further revealed dual mediation pathways influencing FSB: one through grit as an independent mediator, and another through a sequential chain linking grit to reduced anxiety. This foundation enables the exploration of relationships between L2 writing FSB and positive psychological constructs encompassing personality traits, affective states, and institutional factors.

### 2.2. Classroom Psychological Environment and L2 FSB

When discussing educational settings, the term “environment” refers to the general ambiance, tone, climate, or atmosphere of the space (Dorman & Fisher, 2006). The classroom environment is closely linked to the learning motivation, beliefs, and academic performance of students, encompassing aspects such as classroom affinity, cohesion, fairness, and mutual support between teachers and students (Goh & Khine, 2002). In the current study, the components of environment were defined as task orientation, student cohesiveness, and teacher support. Task orientation relates to the importance of completing assignments and staying on topic (Dorman, 2003); student cohesiveness describes the extent to which students know, support, and trust their classmates; and teacher support includes the teacher’s help, relationships, confidence, and enthusiasm toward students (Peng & Woodrow, 2010).

Monteiro et al. (2021) demonstrated that fostering a supportive educational environment directly strengthens students’ sense of school belonging and active behavioral participation. Additionally, such environments indirectly improve academic performance through addressing core psychological requirements, including autonomy, competence, and social connectedness, while simultaneously intensifying intrinsic learning motivation (Reyes et al., 2012). These settings further activate positive affective states, which promote proactive classroom involvement and confident self-expression (H. Wang et al., 2021). Practical implementations, such as structured teacher–student office hours for L2 writing support, further institutionalize FSB through reducing psychological barriers and creating a welcoming climate, as evidenced in Zhou et al.’s (2023) qualitative study. Empirical studies by Zhang (2024) and Zhang and Jiang (2025) have confirmed that teacher support, as an important part of the classroom psychological environment, directly influences FSB. Overall, these findings highlight the classroom psychological environment as a key predictor of students’ FSB in foreign language writing, acting through both direct and indirect pathways.

### 2.3. Grit and L2 FSB

L2 grit, defined as learners’ sustained perseverance and enduring passion toward long-term language goals (Duckworth & Quinn, 2009; Teimouri et al., 2022), remains a subject of structural debate. While initial conceptualizations posited grit as a unitary construct (Duckworth et al., 2007), empirical evidence increasingly supports its dichotomous nature, distinguishing POE (persistent effort despite challenges) from COI (stable enthusiasm over time) (Credé et al., 2017). Divergent findings underscore the need to analyze these dimensions separately. For instance, meta-analytic studies have indicated that POE exerts stronger predictive validity on L2 achievement than COI (Teimouri et al., 2022), while COI demonstrates negligible correlations with affective outcomes, such as foreign language enjoyment (Khajavy & Aghaee, 2024). Such discrepancies suggest that POE and COI may operate through distinct psychological mechanisms, warranting independent examination. To address this theoretical ambiguity and align with recent methodological advancements (Zhao & Wang, 2023a), the current study adopted a two-factor model to disentangle their unique contributions to FSB and test their mediating roles. Rooted in positive psychology, gritty learners exhibit cognitive, affective, and behavioral traits that drive consistent effort and stable interest in learning objectives, thereby enhancing overall engagement (Credé et al., 2017; O’Neal et al., 2018). Specifically, learners with higher POE actively seek, monitor, and internalize writing feedback due to their resilience against setbacks and growth-oriented reframing of criticism (Zhao & Wang, 2023a). COI, though less directly tied to immediate behavioral outcomes, may sustain long-term motivation for iterative feedback cycles.

### 2.4. The Role of FLE as a Mediator Between L2 FSB, Grit, and the Classroom Psychological Environment

FLE has gained prominence as a critical positive emotion in second language acquisition (SLA), influenced by the growing focus on positive psychology (Dewaele & MacIntyre, 2014; Fattahi et al., 2023). Research has extensively explored the determinants of FLE, identifying both external factors, such as teacher personality traits, peer support, and classroom environments, and internal factors, including L2 proficiency, emotion regulation, and L2 grit (Ahmadi-Azad et al., 2020; Liu & Wang, 2021; Pan & Yuan, 2023). Control–value theory (CVT) offers a comprehensive framework for understanding the emergence and impact of achievement emotions such as FLE (Pekrun, 2006). According to CVT, emotions in educational settings stem from learners’ evaluations of control (perceived ability to influence outcomes) and value (subjective significance of tasks). Classroom psychological environments, shaped by teaching practices, peer interactions, and institutional structures, serve as immediate precursors to FLE by shaping these control–value appraisals. For example, constructive teacher feedback bolsters learners’ sense of control over writing outcomes, while cooperative peer dynamics heighten the intrinsic value of language mastery, collectively nurturing FLE (Dewaele & MacIntyre, 2014; Dewaele et al., 2019).

L2 grit, characterized by POE and COI, further refines these cognitive evaluations. POE enhances perceived control through persistent goal-directed action despite obstacles, whereas COI sustains task value by maintaining intrinsic motivation (Liu & Wang, 2021). CVT posits that such appraisals trigger high-arousal, activity-aligned emotions, such as FLE (Pekrun et al., 2010), which subsequently encourage proactive behaviors, such as feedback-seeking behavior (FSB), by mitigating resistance to critical input and reinterpreting feedback as a developmental resource (Ahmadi-Azad et al., 2020). Although prior research confirms independent associations between CVT-aligned antecedents (e.g., classroom environments and grit) and FLE (Pan & Yuan, 2023), the integrative mediation pathway, where FLE connects institutional, trait-based, and behavioral variables, remains understudied. This investigation rigorously examines CVT’s hypothesis that FLE mediates the effects of classroom environments and L2 grit on FSB, thereby unifying environmental, individual, and affective mechanisms within a cohesive theoretical model.

## 3. Methodology

This study investigated Chinese junior secondary students (aged 13–15) enrolled in an English as a foreign language (EFL) writing course. This research aimed to examine the effects of L2 grit, classroom psychological environment, and FLE on FSB, while exploring learner perceptions through a mixed-methods design. A concurrent embedded approach (Creswell & Clark, 2007) prioritized quantitative analysis for hypothesis testing, supplemented by qualitative insights to contextualize the findings.

### 3.1. Participants

In the quantitative phase, 633 junior secondary students (aged 13–15) were recruited through convenience sampling from 8 public schools in northern China. To minimize confounding variables, the participants were selected for homogeneity in their linguistic and educational backgrounds. All of them were monolingual Mandarin speakers. They had more than 5 years of formal English instruction and no overseas exposure. They were in early adolescence, which is marked by heightened environmental sensitivity and emerging self-regulation, making it an ideal age for examining the interplay between L2 grit and FLE. The participants’ reliance on teacher support and peer cohesion, key predictors of FSB, was further amplified by their limited autonomy in structured educational settings.

After excluding 21 invalid surveys (criteria included ≥80% repetitive responses, such as selecting “4” for all items, >10% unanswered questions, or excessively short response times), 612 valid datasets were retained (with a 96.68% validity rate). The sample comprised 342 males (55.88%) and 270 females (44.12%), reflecting regional enrollment trends. All constructs, including the classroom psychological environment (M = 3.52) and L2 grit (M = 3.61), were rated at moderate to high levels on a 5-point Likert scale (range: 3.24–4.16).

For qualitative follow-ups, six 14-year-old students were selected from the valid survey pool using convenience sampling, prioritizing availability, willingness, and comparable English proficiency. To reduce bias, the interviewees had no prior interaction with the research team. All participants (survey respondents and interviewees) were assured confidentiality, voluntary participation, and the right to withdraw without consequences, adhering to ethical protocols.

### 3.2. Instruments

To measure the participants’ classroom psychological environment, L2 grit, FLE, and L2 FSB, this study developed a questionnaire with four scales. Each item was assessed using a 5-point Likert scale, where 1 indicates “strongly disagree” and 5 indicates “strongly agree”. To ensure content validity, the first author translated the English scales into Chinese and shared them with the second author for feedback on phrasing, readability, and meaning.

#### 3.2.1. The Scale for Classroom Psychological Environment

The classroom psychological environment in our study was assessed using a scale adapted from the Classroom Environment Scale (Peng & Woodrow, 2010). This questionnaire was selected for its strong validity and relevance to Asian EFL contexts, where it has been widely validated in capturing three critical dimensions of classroom dynamics: teacher support, student cohesiveness, and task orientation. The scale comprises 13 items divided into three subscales: teacher support (4 items, α = 0.85), student cohesiveness (4 items, α = 0.86), and task orientation (5 items, α = 0.87). Examples of items include: (1) “The teacher provides timely responses to students’ concerns” (teacher support); (2) “I work well with other class members” (student cohesiveness); and (3) “Tasks designed in this class are useful” (task orientation). The instrument’s high internal consistency (α = 0.95 for the full scale in our study) further justified its adoption.

#### 3.2.2. The Scale for L2 Grit

The L2 grit assessment in this study utilized the language-domain-specific Grit Scale (Teimouri et al., 2022), which was tailored for language learning contexts. The scale comprises two dimensions: POE (5 items, α = 0.88) and COI (4 items, α = 0.85), reflecting its domain-specific focus on sustained commitment to long-term language goals. Two samples from the scales are as follows: (1) “I will not allow anything to stop me from my progress in learning English” and (2) “I think I have lost my interest in learning English.” The scale demonstrated strong reliability in this study (α = 0.87), effectively capturing the nuanced dynamics of goal persistence in second language acquisition.

#### 3.2.3. The Scale for FLE

This study employed the culturally sensitive 11-item Chinese Foreign Language Enjoyment Scale (Li et al., 2018), specifically validated for Chinese adolescents’ affective experiences to measure FLE. The scale consists of three parts: FLE—Private (5 items,  α = 0.75), FLE—Teacher (3 items,  α = 0.80), and FLE—Atmosphere (3 items,  α = 0.81). Three samples from the scales are as follows: (1)“I’ve learnt interesting things”; (2) “The teacher is encouraging”; and (3) “There is a good atmosphere.” Demonstrating strong reliability (α = 0.91), the scale’s cultural sensitivity ensures its alignment with the sociocultural nuances of Chinese learners’ emotional engagement, thereby enhancing its ecological validity for this population.

#### 3.2.4. The Scale for FSB in L2 Writing

This study used a scale adapted from the FSB in L2 Writing Scale (Papi et al., 2020). The instrument’s selection was justified by its contextual alignment with academic feedback practices in Chinese tertiary EFL writing instruction, where structured teacher guidance and iterative text revision are central. Previous studies have confirmed its discriminant validity and cross-cultural applicability (Yang & Wang, 2024; Zhang, 2024). The scale comprises two components: feedback monitoring (5 items, α = 0.87) and feedback inquiry (5 items, α = 0.90). These 10 items were selected due to their high internal consistency and ability to assess students’ writing feedback behavior. Two examples from the scales are as follows: (1) “When I get my papers back, I read all of the comments carefully” and (2) “When I do not understand my teacher’s comments on my writing, I ask her/him to clarify.” Its high reliability in this study (α = 0.89) solidifies its psychometric rigor in measuring nuanced L2 learners’ feedback strategies.

#### 3.2.5. Semi-Structured Interviews

The interview protocol had a specific purpose. It aimed to explore two main factors affecting FSB. These factors were the classroom psychological environment and grit. The study especially focused on one aspect—investigating the mediating role of FLE. Building on prior studies examining classroom psychological environments, grit, FLE, and FSB (Li et al., 2018; Papi et al., 2020; Peng & Woodrow, 2010; Teimouri et al., 2022), an interview guide was developed to prompt participants to elaborate on five key themes: (1) the perception and assessment of the classroom psychological environment; (2) the description and function of L2 grit; (3) the experience of FLE during FSB; (4) experiences requesting feedback and strategies for handling it; and (5) the influence of the classroom psychological environment, grit, and FLE on FSB. The first author put together the English guide. Then, the second author checked it for accuracy and clarity. To help Chinese English learners, the first author translated the guide into Chinese. Subsequently, the second author translated it back into English. The research team carefully compared the original English and Chinese versions to make sure the meanings matched.

### 3.3. Quantitative Data Collection and Analysis

After providing their informed consent, the participants filled out a printed questionnaire made by the first author. This took about ten minutes. After collecting the data, all responses were entered into a computerized database for analysis. Standardized procedures ensured the dataset’s integrity and accuracy. Before statistical analysis, the data underwent preliminary screening. To address the first research question, confirmatory factor analysis (CFA) was carried out. This validated the measurement models for the classroom psychological environment, L2 grit, FLE, and FSB. Then, descriptive statistics and correlational analyses were performed using SPSS 26. Finally, structural equation modeling (SEM) with maximum likelihood estimation in AMOS 26 was used to determine the direct effects of the classroom environment and L2 grit on FM and FI, and the mediating role of FLE. The bootstrap method was used to assess the mediating effect’s significance.

### 3.4. Qualitative Data Collection and Analysis

Semi-structured interviews were employed to gain nuanced insights into the participants’ individual experiences and perceptions. During these sessions, the participants were asked about their classroom environment perceptions, English writing grit, FLE, and how these factors influenced their FSB. Face-to-face interviews lasting approximately 20 min each were audio-recorded by the first author and transcribed verbatim. The second author independently verified the transcription accuracy.

The qualitative data analysis followed an inductive, recursive process comprising three systematic phases. First, both researchers independently reviewed the interview transcripts to identify recurring terms and phrases related to classroom environment, grit, FLE, and FSB. Open coding generated 84 initial codes (e.g., the avoidance of direct feedback inquiry to maintain hierarchical harmony), which were subsequently grouped into 10 categories through axial coding, exploring shared characteristics and interconnections (e.g., other influencing factors in feedback-seeking). Theoretical coding then synthesized these categories into five overarching themes, (e.g., the function of the classroom environment, L2 grit, and FLE in FSB) by identifying the latent relationships among the constructs. Crucially, the coding framework explicitly integrated cultural dimensions salient to Chinese learners (e.g., respect for authority) to ensure culturally grounded interpretations. To ensure rigor, intercoder reliability was established through dual independent coding of 20% of the transcripts, achieving 90% agreement. Discrepancies were resolved via consensus discussions, with unresolved cases arbitrated by a third researcher, thereby safeguarding the trustworthiness and depth of the thematic framework. Due to the study’s primary focus on quantitative analysis, a condensed coding scheme is provided (see Table 1).

## 4. Quantitative Results

### 4.1. Confirmatory Factor Analysis

Prior to data analysis, data screening was carried out, and neither multicollinearity nor normality assumptions were broken. To assess how well the empirical data matched each important component, six CFA models were employed. For the classroom psychological environment, the model fit was satisfactory (x^2^/df = 1.98, CFI = 0.99, TLI = 0.99, RMSEA = 0.04, and SRMR = 0.02). The model fit was also satisfactory for POE (x^2^/df = 2.78, CFI = 0.99, TLI = 0.99, RMSEA = 0.05, and SRMR = 0.02). For COI, the model fit was acceptable (x^2^/df = 4.87, CFI = 0.99, TLI = 0.98, RMSEA = 0.08, and SRMR = 0.02). The model fit for FLE was also satisfactory (x^2^/df = 1.49, CFI = 0.99, TLI = 0.99, RMSEA = 0.03, and SRMR = 0.02). For FM in L2 writing, the model fit was acceptable (x^2^/df = 4.64, CFI = 0.99, TLI = 0.97, RMSEA = 0.08, and SRMR = 0.02). Lastly, an acceptable model fit was found for FI in L2 writing (x^2^/df = 4.73, CFI = 0.99, TLI = 0.98, RMSEA = 0.08, and SRMR = 0.02).

### 4.2. Descriptive and Correlation Analyses

The participants exhibited moderate to high levels across key constructs: classroom psychological environment (M = 3.38, SD = 1.12), FLE (M = 3.41, SD = 1.22), POE (M = 3.39, SD = 1.11), COI (M = 3.49, SD = 1.06), and FM (M = 3.39, SD = 1.11). The FI scores were comparatively lower (M = 2.93, SD = 1.17), indicating a preference for passive feedback monitoring over active inquiry. Bivariate correlations (Table 2) revealed statistically significant positive relationships among all variables (*p* < 0.01). Notably, classroom environment correlated moderately with FLE (r = 0.274), POE (r = 0.241), and COI (r = 0.251), supporting its foundational role in shaping both grit and affective states. FLE showed strong associations with FM (r = 0.466) and FI (r = 0.515), underscoring its pivotal role in mediating FSB. POE and COI exhibited moderate correlations with FM (r = 0.417) and (r = 0.431), respectively, consistent with grit’s emphasis on sustained effort and stable interest. However, their weaker correlations with FI, (r = 0.306) and (r = 0.275), underscore cultural and motivational barriers to proactive inquiry.

### 4.3. Structural Equation Modeling

**Figure 1:**
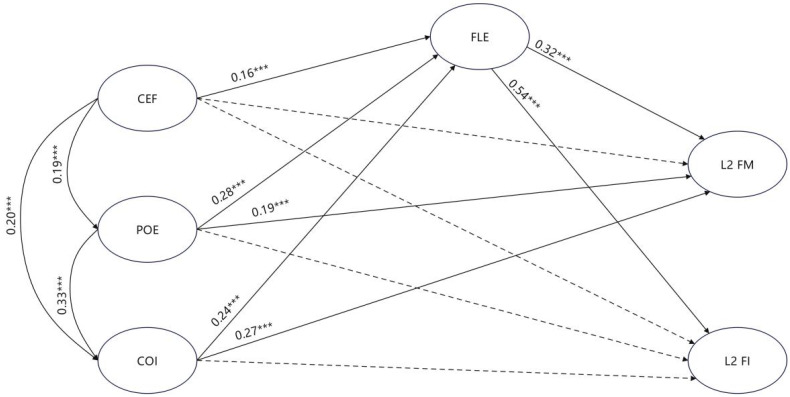
Standardized coefficients among classroom psychological environment, FLE, L2 grit, and FSB. Note: CEF = classroom psychological environment; FLE = foreign language enjoyment; POE = perseverance of effort; COI = consistency of interest; FM = feedback monitoring; and FI = feedback inquiry. *** *p* < 0.001.

The questionnaire data validated the expected SEM model (see Figure 1), as evidenced by its acceptable model fit (x^2^/df = 1.31, *p* < 0.001, CFI = 0.98, TLI = 0.98, RMSEA = 0.02, and SRMR = 0.03). The path diagram illustrates the standardized coefficients for hypothesized relationships. Solid lines denote significant paths, while dashed lines indicate non-significant direct effects.

Classroom environment significantly predicted FLE (β = 0.16, *p* < 0.001) but showed no direct effects on FM (β = 0.05, *p* = 0.21) or FI (β = 0.09, *p* = 0.03), suggesting its influence operates indirectly via affective pathways. POE directly enhanced FLE (β = 0.28, *p* <  0.001) and FM (β = 0.19, *p* <  0.001), while COI predicted FLE (β = 0.24, *p* < 0.001) and FM (β = 0.27, *p* < 0.001). Neither grit dimension directly influenced FI (*p* > 0.05), highlighting the necessity of FLE as a mediator. FLE strongly predicted both FM (β =  0.32, *p* < 0.001) and FI (β =  0.54, *p* < 0.001), confirming its central mediating role.

The indirect effect was further deconstructed to reveal the mediator’s role. The findings demonstrate that, through the FLE mediator, the classroom psychological environment indirectly improved L2 FM (β = 0.05, 95% CI = [0.02, 0.10], *p* = 0.001) and L2 FI (β= 0.09, 95% CI = [0.03, 0.16], *p* = 0.003). The findings demonstrate that POE, mediated by FLE, indirectly enhanced L2 FM (β = 0.09, 95% CI = [0.04, 0.18], *p* < 0.001) and FI (β = 0.15, 95% CI = [0.07, 0.26], *p* < 0.001). In contrast, while the indirect effects of COI on FM and FI were slightly weaker (FM: β = 0.08, 95% CI = [0.03, 0.14], *p* = 0.003; FI: β = 0.13, 95% CI = [0.04, 0.23], *p* = 0.004), they remained significant.

## 5. Qualitative Findings

### 5.1. Efficacy in Teacher Support but Different Views on Student Cohesiveness

All respondents highlighted the effectiveness of teacher support in promoting L2 FSB. For instance, S1 noted, “The teacher always asks me privately about my writing struggles and gives step-by-step suggestions. This makes me feel valued, so I’m more willing to ask for feedback”. Similarly, S3 emphasized, “After the teacher praised my essay structure, I felt confident to submit drafts early for comments”. Quantitative data corroborated this: teacher support scored highest (M = 4.2/5) in classroom environment ratings, aligning with the interview themes.

However, the students’ views on peer cohesion diverged. Four interviewees viewed peer cohesion positively, with S6 stating, “We form study groups to compare teacher feedback. Seeing others’ mistakes helps me avoid them”. In contrast, S4 criticized excessive cohesion, “Group discussions waste time. Peers’ advice feels enough, so I skip asking the teacher”. This divergence suggests that while cohesiveness enhances FSB for some by building communal support (e.g., through shared accountability and peer encouragement), it inadvertently limits teacher-directed inquiry for others due to complacency or perceived inefficiency in balancing peer and teacher interactions.

### 5.2. Feedback Monitoring Preferred over Inquiry

Most students tend to carefully monitor their teachers’ feedback rather than actively seek further clarification. This phenomenon is driven by two primary factors: differences in self-perceived language competence and deeply ingrained cultural norms. Students with weaker English writing skills generally lack confidence in their abilities and fear that asking questions might expose their perceived shortcomings. S1, a low-proficiency learner, explained this avoidance: “I fear asking questions will expose my poor grammar. It’s safer to silently note corrections”. In contrast, more proficient students are inclined to engage proactively. S3 demonstrated proactive FI: “After each essay, I discuss feedback with the teacher to refine my arguments, and this builds my confidence”.

Cultural reverence for authority further reinforces this behavioral pattern. Students widely regard teachers as the primary source of expertise, whose feedback is seen as unquestionable guidance. S2 articulated this norm: “Questioning feedback publicly feels disrespectful. I wait to ask privately if needed”. S5 noted, “I revise drafts thoroughly based on comments but avoid direct inquiries to maintain harmony”. In Chinese educational contexts, public questioning might be perceived as disrespectful to teachers or disruptive to collective harmony. In summary, the divergence in feedback-seeking behaviors arises not only from individual competence disparities but also from deeper cultural values. The interplay between low self-efficacy and respect for authority solidifies passive monitoring as the default choice for most students, even among those with strong learning motivations.

## 6. Discussion

### 6.1. The Collective Predictive Roles of L2 Grit and Classroom Environment on FSB in L2 Writing

The quantitative findings comprehensively address Research Question 1. They demonstrate that L2 grit (POE and COI) and classroom psychological environment collectively predict FSB in L2 writing. However, they do so differentially, through distinct pathways. Crucially, the classroom psychological environment showed no direct effects on FSB, contrasting with prior studies that have emphasized its standalone impact (Ma & Wei, 2022; M. T. Wang et al., 2020). Instead, its influence operated entirely through indirect affective pathways, aligning with the control–value theory (Pekrun, 2006), which posits that environmental factors shape emotions that drive behavioral outcomes. This underscores the classroom environment’s foundational yet indirect role as a catalyst for feedback engagement.

Meanwhile, L2 grit dimensions (POE and COI) exhibited direct and distinct predictive effects on FM. Learners high in POE demonstrated relentless persistence in refining their writing and attentiveness to feedback (Pekrun, 2006). Conversely, those with high COI displayed intrinsic curiosity and engagement, motivating active feedback monitoring as a growth tool (Vansteenkiste et al., 2004). However, neither POE nor COI directly predicted feedback inquiry (FI), highlighting a critical boundary condition where grit and classroom environment collectively but incompletely shape FSB. While grit fosters persistence and interest, it is insufficient to overcome FI’s inherent demands; specifically, proactive dialogue requiring cultural adaptability (Yao & Zhu, 2024). This divergence reveals that grit and classroom environment collectively (but incompletely) shape FSB, as FI remains constrained by external factors, such as cultural norms.

### 6.2. Mediating Mechanisms of FLE in Bridging L2 Grit, Classroom Environment, and FSB

The SEM results elucidate how FLE mediates the relationships among L2 grit, classroom environment, and FSB. Specifically, FLE fully mediated the effects of classroom environment on both FM (β = 0.05) and FI (β = 0.09). This indicates that a supportive classroom climate enhances students’ enjoyment, which in turn motivates proactive feedback engagement, highlighting FLE’s critical role as an affective conduit. For L2 grit dimensions, FLE partially mediated their influence on FM (β = 0.13), amplifying the effects of POE and COI. Qualitative findings further differentiated these pathways: POE-driven learners focused on identifying textual deficiencies, whereas COI-driven learners prioritized seeking constructive advice, both sustained by enjoyment rooted in perceived growth. However, neither grit dimension significantly predicted FI, underscoring that proactive inquiry requires additional factors (e.g., cultural adaptability) beyond grit and FLE.

These mechanisms align with the control–value theory (Pekrun, 2006), wherein FLE, as a positive emotion, reallocates cognitive resources and enables adaptive strategies. The broaden-and-build theory (Isgett & Fredrickson, 2004) explains how FLE broadens cognitive flexibility for exploratory behaviors such as FSB, though cultural barriers (e.g., “face” concerns) may constrain this effect. In summary, FLE acts as both an affective conduit (translating classroom support into feedback engagement) and an amplifier (enhancing grit’s impact on FM). This dual role underscores FLE’s centrality in integrating contextual, personal, and behavioral dynamics in L2 writing.

### 6.3. Cultural Constraints on FI

Qualitative studies have shown that cultural norms, particularly respect for authority and the preservation of “face”, critically influence learners’ FSB. First, hierarchical cultural values equate student-initiated dialogue with disrespect toward authority figures (Chan & Smith, 2024), framing FI as a disciplinary infraction rather than a learning opportunity. This norm persists even in supportive classroom environments. English remains institutionalized as an academic subject, rather than a communicative tool (Long & Zhang, 2022). This reinforces a unidirectional feedback culture (teacher-to-student). Such a culture stigmatizes FI as inappropriate. Consequently, students prioritize compliance over proactive inquiry to align with sociocultural expectations, silencing themselves to avoid perceived challenges to instructors’ authority.

Second, the “face” culture amplifies this suppression by conflating public feedback-seeking with reputational risk. FI inherently demands public dialogue, which threatens to expose perceived shortcomings and violate norms of social harmony, a core tenet of “face” preservation (Hu et al., 2024). Even learners with high grit suppress FI to prioritize collective dignity over individual growth, fearing embarrassment or damage to their social reputation. This creates a dual suppression effect: while POE and COI enhance FM, they fail to predict FI due to cultural barriers that isolate FI from grit’s motivational reach. In summary, cultural norms act as a sociocultural filter, selectively permitting FM (a private, low-risk behavior) while suppressing FI (a public, high-stakes action). This dynamic underscores the need to mitigate the effects of authority and “face” norms on equitable feedback engagement in L2 contexts.

## 7. Conclusions

This study explored how the classroom psychological environment, L2 grit, and FLE interact to influence FSB in L2 writing. The results show that the classroom environment indirectly boosts FM and FI by increasing FLE, rather than directly driving feedback-related actions. POE and COI in L2 grit directly enhance FM, with FLE mediating their indirect impact on FM. However, neither POE nor COI directly affects FI. The qualitative data also show that Chinese cultural norms, such as “face-saving” and respect for authority, hinder proactive feedback-seeking. Even high-grit learners tend to favor non-confrontational FM. To address this, institutional support should consider cultural factors. Overall, the findings highlight the need for environmental and individual factors to work together via affective mechanisms to achieve behavioral outcomes.

Building on these insights, actionable strategies emerge to optimize L2 writing instruction in Chinese EFL contexts. First, educators should cultivate psychologically safe classrooms by integrating structured peer reviews, anonymous feedback platforms, and teacher–student office hours, thereby mitigating cultural barriers such as “face-saving”. To empower teachers as agents of change, professional development programs should prioritize scaffolded questioning techniques and peer-led Q&A workshops, equipping educators to guide students in transitioning from passive feedback acceptance to proactive inquiry. Second, curriculum designers must embed grit-enhancing tasks, such as long-term iterative writing projects and thematic assignments aligned with student interests, in order to foster perseverance and sustained motivation. Teachers can reinforce these goals through goal-setting workshops and self-reflection exercises, helping students to link feedback to long-term language mastery. Finally, leveraging FLE as a catalyst for intrinsic motivation requires enthusiastic teacher demeanors, a recognition of incremental progress, and metacognitive tools, such as feedback portfolios. Through the integration of these strategies, educators harmonize institutional support with learner agency, transforming students from passive recipients into active collaborators in their language learning journeys.

While this study provides critical insights, its limitations necessitate further exploration. First, the focus on junior high students limits its generalizability. Longitudinal studies tracking FSB across developmental stages could reveal how grit and FLE evolve with cognitive maturity. Second, the sample’s male-skewed gender ratio (55.88%) may influence the findings through two pathways: gender may moderate the impact of psychological variables on writing performance. Females typically outperform males (Yao et al., 2024), which could bias group-level analyses toward male patterns. Potential gender differences in feedback preferences (e.g., male tendencies toward specific types) could alter variable associations (Berlin & Dargnies, 2016). Therefore, future studies should perform gender subgroup comparisons. Third, comparative studies across diverse educational systems (e.g., Western vs. Confucian-heritage contexts) could disentangle universal and culture-bound factors influencing FSB. Addressing these gaps will help to refine theoretical models and inform culturally adaptive teaching practices.

## Figures and Tables

**Table 1:** Coding scheme for qualitative data analysis.

Code	Category	Theme
Academic guidance from the teacher, teachers’ individualized instruction, encouragement, teamwork, and peer review	Teacher support, student cohesiveness, and other aspects of English writing	Various aspects of classroom environment
Deep interest in L2 learning, great enthusiasm for L2 learning, and eagerness for L2 learning	Constancy of interest related to L2 learning	Grit in foreign language learning
Invest a lot of energy and time in L2 learning, make the utmost effort in L2 learning, and never give up when met with difficulties	Perseverance of effort related to L2 learning	
Be willing to ask for feedback, more likely to seek feedback, and obtain pleasure from feedback-seeking	Foreign language enjoyment related to feedback-seeking	Foreign language enjoyment
Read the feedback, review the feedback, and keep track of the feedback	The use of feedback monitoring	FSB in L2 writing
Obtain additional feedback, request clarification, and converse with the teacher about the feedback received	The use of feedback inquiry	
Phrases or expressions describing the connections among the important factors	Effect of classroom environment on feedback-seeking	The function of the classroom environment, L2 grit, and FLE in FSB
	Effect of grit on feedback-seeking	
	Effect of foreign language enjoyment on feedback-seeking	
	Other influencing factors in feedback-seeking	

**Table 2:** Descriptive statistics and bivariate correlations at the factor level.

Latent Variables	M	SD	1	2	3	4	5	6
1. Classroom psychological environment	3.38	1.12	1					
2. Foreign language enjoyment	3.41	1.22	0.274 **	1				
3. Perseverance of effort	3.39	1.11	0.241 **	0.379 **	1			
4. Consistency of interest	3.49	1.06	0.251 **	0.369 **	0.431 **	1		
5. Feedback monitoring	3.39	1.11	0.245 **	0.466 **	0.417 **	0.441 **	1	
6. Feedback inquiry	2.93	1.17	0.247 **	0.515 **	0.306 **	0.275 **	0.431 **	1

Note: ** *p* < 0.01. The same applies hereinafter.

## Data Availability

The data presented in this study are available upon request from the corresponding author.
